# Epidemiology of Risk Stratification, Machine Learning Early Prediction Model, and Tumor Suppressive Mechanism of RHBDF2 in Esophageal Cancer in Gansu Province

**DOI:** 10.1002/cam4.71605

**Published:** 2026-03-17

**Authors:** Duojie Zhu, Yinggang Che, Huijuan Cheng, Weiqi Wang, Yumin Li

**Affiliations:** ^1^ Department of Thoracic Surgery, The Second Hospital & Clinical Medical School Lanzhou University Lanzhou China; ^2^ Department of Respiratory Medicine Air Force Hospital of Western Theater Command, PLA Chengdu China; ^3^ The Second Hospital & Clinical Medical School Lanzhou University Lanzhou China; ^4^ Department of Oral and Maxillofacial Surgery, School of Stomatology Fourth Military Medical University Xi'an China; ^5^ Gansu Province Key Laboratory of Environmental Oncology Lanzhou University Lanzhou China

**Keywords:** diet, esophageal cancer epidemiology, lifestyle, primary prevention, RHBDF2

## Abstract

**Background:**

Esophageal cancer imposes a considerable health burden in the high‐risk areas of Northwest China, necessitating the development of effective biomarkers for its early detection to address this public health challenge.

**Methods:**

We integrated multi‐omics analysis, machine learning algorithms, and population epidemiology investigations in Gansu Province to screen and develop biomarkers for the early detection of esophageal cancer.

**Results:**

Epidemiological findings revealed distinct geographical variations in esophageal cancer incidence, with Yugu County being the sole high‐risk area (incidence rate: 54.2/100,000). Cases were predominantly in individuals aged > 40 years; males were the main affected population except in Yugu County. Protective factors for the disease included a healthy diet, regular exercise, and positive emotions, while smoking, alcohol consumption, and high‐salt intake were identified as risk factors. A random forest machine learning model exhibited excellent predictive performance (AUC = 0.995) and identified key predictive factors for esophageal cancer. Proteomic analysis further revealed that RHBDF2 was downregulated and could serve as a potential biomarker for the disease.

**Conclusions:**

This study provides robust epidemiological and molecular evidence for esophageal cancer prevention and early intervention strategies, and the identified potential biomarker RHBDF2 and high‐performance predictive model offer valuable tools for the early detection of esophageal cancer in high‐risk regions of Northwest China.

## Graphical abstract

(A) Epidemiological Stratification, Risk Modeling, and Molecular Mechanisms: RHBDF2 Inhibits Esophageal Cancer Progression via EMT and KRAS Signaling in Gansu's Risk‐Stratified Regions. (B) Schematic Workflow of the Study: From Clinical Risk Assessment to Molecular Mechanisms in Esophageal Carcinoma.

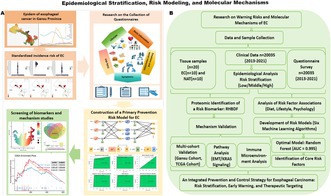

**Abbreviations**
ASR: age‐standardized prevalence rates
AUC: area under the curve
DEG: differentially expressed genes
EMT: epithelial mesenchymal transition
ESCA: esophageal cancer
FDR: false discovery rate
GO: gene ontology
GSEA: gene set enrichment analysis
KEGG: Kyoto encyclopedia of Genes and Genomes
KRAS: Kirsten rat sarcoma viral oncogene homolog
log2FC: log2 fold change
PCA: principal component analysis
ROC: receiver operating characteristic
TCGA: the Cancer Genome Atlas

## 1 Introduction

Esophageal cancer is a significant global public health issue, owing to its aggressive characteristics and poor prognosis. It ranks among the top 10 cancers worldwide, with approximately 511,000 new cases and 445,000 deaths reported in 2022. Despite the advancements in medical interventions, the 5‐year survival rate of esophageal cancer remains low, at approximately 20% [1, 2], underscoring the urgent need to enhance the prevention and treatment strategies [3].

As one of the most prevalent malignancies worldwide, esophageal cancer imposes a severe burden in China, where more than 53% of global cases of esophageal squamous cell carcinoma are reported [4]. The Gansu Province is a high‐incidence region [5]. The incidence rates of esophageal cancer vary significantly across different regions, closely linked to a multitude of factors such as geographic location, lifestyle, dietary habits, and healthcare conditions.

The etiology of esophageal cancer in Gansu Province is complex, involving a combination of genetic susceptibility and environmental exposures, including coal smoke, malnutrition, consumption of hot beverages, and certain fungi [6, 7, 8]. Smoking and excessive alcohol consumption are recognized risk factors globally [9, 10, 11]. Given this diverse array of risk factors, a comprehensive understanding and mitigation of the impact of esophageal cancer is crucial for the development of effective primary prevention strategies. Primary prevention is vital for controlling and reducing the incidence of esophageal cancer [12, 13], particularly because this malignancy is often diagnosed at advanced stages [14]. Therefore, further research is needed to enhance our understanding of the mechanisms underlying the development of esophageal cancer and to improve early detection strategies.

This study, conducted between 2013 and 2021, examined the incidence of esophageal cancer in 10 Gansu districts/counties. It explores the regional trends, influencing factors, and mechanisms, offering epidemiological insights that can enhance awareness, optimize resources, inform policy‐making, and offer practical applications (Graphical abstract).

## 2 Methods and Materials

### 2.1 Data Acquisition and Sample Source

The clinical data (2013–2021) were obtained from Gansu Provincial Health Commission's Esophageal Cancer Health Big Data, including 20,035 diagnosed patients. Overall, 10 representative counties in Gansu (covering Han, Dongxiang, Baoan, Hui, Tibetan, Yugu ethnicities) were selected to investigate the influences of ethnicity, diet, and psychology. A survey (July 2021–June 2022) enrolled 2129 individuals and the data were extracted from Gansu Big Data Monitoring System. These regions represented 10.1% of Gansu's population and 11.67% of the 2021 esophageal cancer cases, with monitoring stations established for further research.

### 2.2 Population Standardization of Esophageal and Gastric Cancer Incidence in Gansu Province

Using the 2008 Gansu census as the standard, the populations were categorized into 19 age groups. The district/county population data (2021) from Gansu Statistics Bureau and esophageal/gastric cancer cases were used for calculations (data request URL: http://tjj.gansu.gov.cn/tjj/c109529/mail_detail.shtml?id=fa6977628d3145e0b0f027be5b6efc9a). The age‐standardized prevalence rates (ASR) were computed as ∑(age‐specific cases/population*standard population percentage).

Esophageal cancer ASR was stratified using the 2019 Xinxiang Consensus per 100,000 population as < 5 (low), 5–15 (medium), > 15 (high), and > 50 (extremely high). Gastric cancer ASR was stratified using the 2022 Beijing Guidelines per 100,000 population as < 10 (low), 10–20 (medium), 20–35 (high), and > 35 (extremely high).

### 2.3 Design and Acquisition of the Esophageal Cancer Patient Questionnaire

A field survey (Supporting Information 1), conducted via questionnaires at targeted sites to investigate esophageal cancer risk factors, was approved by the Medical Ethics Committee of the Second Hospital of Lanzhou University (2024A‐059). The questionnaire included 64 characteristics across six categories: baseline data, lifestyle habits, dietary traits, emotional/psychological factors, clinical symptoms, and comorbidities. A total of 2129 patient questionnaires were collected to analyze their potential association with the risk of esophageal cancer.

### 2.4 Establishment of a Primary Risk Model for Esophageal Cancer

Questionnaires from 2129 patients were preprocessed; after excluding samples with missing data, 1203 respondents remained, of whom 926 with 64 indicators were included for modeling. The indicators were standardized, and the patients were categorized into training (70%) and testing (30%) cohorts. Using R packages (“VIM”, “randomForest”, “caret”), six risk models were constructed: recursive partitioning/regression trees, random forest, naive Bayes, support vector machine, K‐nearest neighbors, and backpropagation neural network. Model performance in the testing cohort was evaluated via accuracy and ROC‐AUC to select the optimal model. The key risk factors and their interconnections were analyzed using the “randomForestExplainer” function in R.

### 2.5 Protein Sequencing

Esophageal tissues were lysed in sodium dodecyl sulfate‐tris–HCl buffer, homogenized (homogenizer, 24 cycles*2, 6.0 m/s, 60 s), sonicated, and boiled. After centrifugation (14,000 *g*, 15 min), the supernatants were filtered (0.22 μm) and quantified. For filter aided ample preparation, 200 μg protein was reduced (dithiothreitol, 100 mM), alkylated (iodoacetamide, 100 mM), and digested with trypsin (4 μg, 37°C, 16–18 h) following buffer exchange (urea buffer/triethylammonium bicarbonate buffer). Peptides were desalted (C18), lyophilized, and fractionated using high‐pH reverse phase high performance liquid chromatography (Agilent 1260, XBridge C18, 5%–45% acetonitrile over 40 min, 36 fractions merged into 12).

Data dependent acquisition was performed on a Q‐Exactive HF‐X coupled to an Easy Nano Liquid Chromatography system (300 nL/min, non‐linear gradient: 1% B for 0–5 min, 1%–28% B for 5–95 min, 28%–38% B for 95–110 min, 38–100% B for 110–115 min, 100% B for 115–120 min; electrospray 2.0 kV). The mass spectrometry parameters were as follows: *m/z*, 350–1500; resolution, 60,000 and 15,000; and normalized collision energy, 28%. Data dependent acquisition included dynamic exclusion (30 s). Raw data were searched (Spectronaut v12) against a custom database (trypsin, ≤ 2 missed cleavages; fixed carbamidomethyl‐C, variable oxidation‐M/N‐terminal acetylation; 1% FDR for precursors/proteins/peptides). Data independent acquisition was performed using an automatic gain control of 2 × 10^5^ (vs. 1*10^5^ for data dependent tandem mass spectrometry) and 50 ms max injection time (vs. 30 ms).

### 2.6 Quality Control

Overall, 20 qualified datasets were filtered via standard proteomic pipelines. The quality controls included peptide mass error ±10 ppm, peptide/protein FDR ≤ 0.01 to reduce noise. The data quality was validated by peptide score clustering, consistent intra‐group expression, high sample correlation, and distinct PCA separation between groups (Supporting Information 2 [Figure S1A–D]).

### 2.7 Differential Analysis

To screen prognostic molecules across esophageal cancer risk levels, 20 samples (10 cancer, 10 controls) from different risk regions of Gansu (3 low/medium/high‐risk each) were used for proteomic sequencing. The study was approved by the Medical Ethics Committee of the Second Hospital of Lanzhou University (No. 2024A‐059) and written informed consent was provided by the participants. RHBDF2 was identified as a prognostic molecule via pairwise differential screening. In TCGA‐ESCA, the samples were divided into RHBDF2 high‐ and low‐expression groups (50% cutoff). Differentially expressed genes (DEGs) were identified using the limma package (|log2 fold change| > 0.5, *p* < 0.05). Volcano plots and heatmaps of top DEGs were generated via ggplot2 and pheatmap. Pathway enrichment was analyzed with “clusterProfiler”. DEG intersections from pairwise comparisons were analyzed using jvenn to obtain candidate genes.

### 2.8 Immune Microenvironment Analysis

To investigate the changes in the ESCA immune microenvironment between the RHBDF2 high‐risk and low‐risk groups, the xCell algorithm (*p* < 0.05) was used to analyze the content and relative abundance of 64 types of immune infiltrating cells and other cell types in the TCGA cohort. Additionally, the Wilcoxon test (*p* < 0.05) was applied to analyze the differences in immune cell infiltration between the high‐risk and low‐risk groups, and the results were presented in the form of boxplots.

## 3 Gene Set Enrichment Analysis (GSEA)

In the TCGA ESCA cohort, to explore the pathways that may be enriched by DEGs between the two RHBDF2 risk groups, the Limma package was used to calculate the log2 fold change (log2FC) of DEGs between the two risk groups. Subsequently, the genes were sorted in descending order based on log2FC to generate a gene list associated with each prognostic gene. Subsequently, based on background gene sets (h.all.v7.1.symbols.gmt, c2.cp.kegg.v7.1.symbols.gmt, etc.) from the Molecular Signatures Database (MSigDB; https://www.gsea‐msigdb.org/gsea/msigdb), the clusterProfiler package was used to perform GSEA on the DEGs.

### 3.1 Other Statistical Analyses

Data Preprocessing, Presentation, Sample Size Specification, and Statistical Methods.

#### 3.1.1 Questionnaire Data

The missing values were handled using the multiple imputation method. The outliers were identified via box plots; after verification, those confirmed as entry errors were corrected. Continuous variables (e.g., age, body weight, dietary intake) were standardized with *Z*‐score normalization to satisfy the normality requirement for model construction.

#### 3.1.2 Proteomics Data

Data filtering was performed using Spectronaut v12 software; spectra whose peptide mass error exceeded ±10 ppm were excluded. Reliable peptides and proteins were screened based on the 1% false discovery rate (FDR) criterion. Proteins with a missing value proportion > 30% were removed, and the remaining missing values were imputed via the *K*‐nearest neighbor algorithm. A principal component analysis (PCA) was applied to verify data quality, ensuring distinct intragroup sample clustering and intergroup sample separation.

#### 3.1.3 Data Presentation

Continuous data (e.g., age, body weight, pulse rate) were expressed as “mean ± standard deviation (mean±SD)”. Categorical data (e.g., sex distribution, lifestyle categories) were presented as “frequency (percentage) [*n* (%)]”. The expression levels of DEGs were denoted as log2FC, while the protein expression levels were represented by normalized intensity values.

#### 3.1.4 Sample Size Specification

For epidemiological analysis, the clinical data sample size was 20,035 (patients confirmed with esophageal cancer) from 2013 to 2021; the questionnaire survey involved 2129 samples, with 1203 valid samples after preprocessing, among which 926 were used for modeling (training set *n* = 648, test set *n* = 278). For proteomics analysis, 20 sequencing samples were included (*n* = 10 esophageal cancer tissues and *n* = 10 normal esophageal tissues), with approximately three samples collected from each of the three low/medium/high‐risk regions to ensure the representativeness of risk stratification. For the TCGA‐ESCA cohort analysis, the RHBDF2 high‐expression and low‐expression groups contained 99 samples each, respectively (divided by the 50% cutoff). For immune microenvironment analysis and gene set enrichment analysis (GSEA), the sample size of the TCGA and ESCA cohorts was 185 samples each.

#### 3.1.5 Detailed Statistical Methods

For comparisons between the two groups, the independent‐samples *t*‐test (two‐tailed) was adopted for continuous data with normal distribution and homogeneous variances; otherwise, the Wilcoxon rank‐sum test (two‐tailed) was used. For comparisons between multiple groups (> 3 groups), the Kruskal–Wallis test was used. The chi‐square test (*χ* ^2^ test, two‐tailed) was applied for categorical data. For comparisons among multiple groups, a one‐way analysis of variance (ANOVA) was used for continuous data, followed by Tukey's test for multiple comparisons. DEGs/proteins were screened using the limma package with the thresholds of |log2FC| > 0.5 and *p* < 0.05 (two‐tailed test), and FDR correction was employed to control the false positive rate. Six algorithms, including random forest and support vector machine, were used for model construction and evaluation; the model parameters were optimized via 10‐fold cross‐validation, and model performance was assessed using the area under the curve (AUC) and accuracy, with the significance level *α* = 0.05. The Pearson correlation analysis was used for bivariate normally distributed data, while Spearman's rank correlation analysis was applied for non‐normally distributed data. The Shapiro–Wilk test was used for the normality test and Levene's test for homogeneity of variance test to ensure that the prerequisite assumptions of the selected statistical methods were met. For small sample data (e.g., *n* = 20 for proteomics sequencing), a combination of normality transformation and nonparametric tests was adopted to improve the reliability of results.

Statistical Software: A data analysis was conducted using R version 4.3.3 (R Foundation for Statistical Computing, Vienna, Austria), with related packages including VIM, randomForest, caret, limma, clusterProfiler, pheatmap, and ggplot2. Proteomics data processing was performed using Spectronaut v12 (Biognosys, Schlieren, Switzerland). Graphs were plotted using R version 4.3.3 and GraphPad Prism 9.0 (GraphPad Software, San Diego, USA).

## 4 Results

### 4.1 Epidemiological Analysis in Gansu Province (2013–2021)

The esophageal cancer incidence across 10 Gansu counties (Supporting Information 2 [Figure S2A]) showed distinct trends: Jishishan, Zhouqu, and Yugu had increasing rates; Wudu, Yuzhong, Dongxiang, Zhangjiachuan, Minqin, and Lintao increased and subsequently declined (Wudu fluctuated notably 2016–2019); and the rates in Gaolan remained stable.

Minqin had the highest 9‐year case count, followed by Wudu and Yuzhong, with Yugu the lowest (Supporting Information 2 [Figure S2B]). Incorporating the 2021 census data (Supporting Information 2 [Figure S2C]), Yugu had the highest incidence (54.224/100,000), while Minqin (36.636/100,000) and Yuzhong reported the lowest (2.951/100,000; Supporting Information 2 [Figure S2D]). Minqin reported the highest prevalence (439.064/100,000), followed by Yugu and Zhouqu, while Yuzhong, Jishishan, and Lintao reported the lowest (Supporting Information 2 [Figure S2E]). These results highlight substantial county‐level disparities, necessitating localized incidence/prevalence studies.

### 4.2 Esophageal Cancer Risk Area Assessment and Classification

**FIGURE 1:**
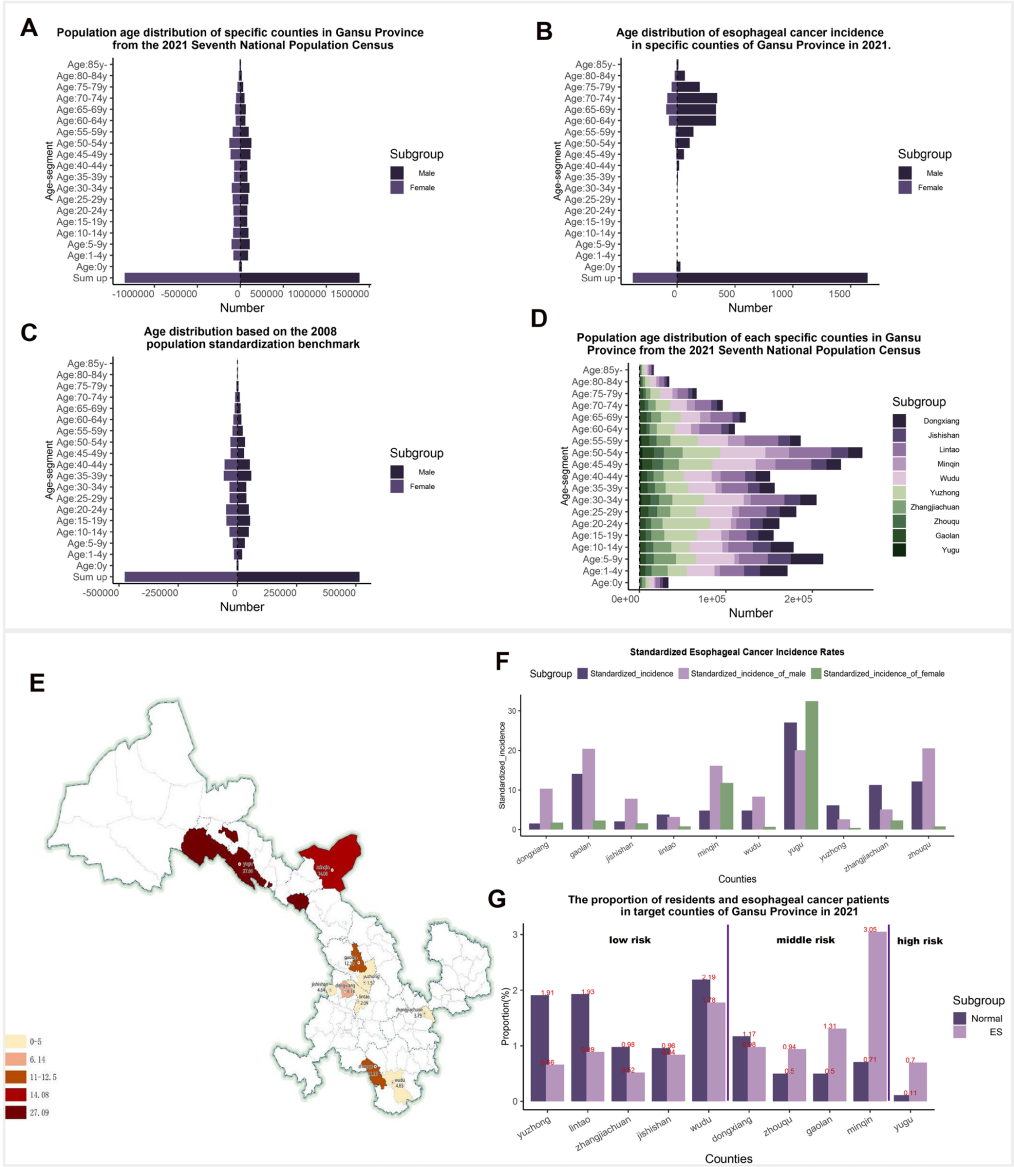
(A) Age distribution of populations across Gansu counties based on the 2021 Seventh National Population Census. (B) Age distribution of esophageal cancer incidence in selected Gansu counties for 2021. (C) Age distribution referenced to the 2008 population standardization benchmark. (D) Stacked bar chart showing population composition ratios across Gansu study counties based on the 2021 Seventh National Population Census. (E) Geographic distribution map of standardized esophageal cancer risk levels across Gansu study counties. (F) Bar chart showing differences in standardized esophageal cancer incidence rates between overall, female, and male populations across Gansu study counties. (G) Bar chart showing the proportion of general population vs. esophageal cancer patients across Gansu study counties stratified based on risk level.

The risk assessment and classification of esophageal cancer regions are critical for targeted public health strategies. We generated an age‐stratified population distribution map using the seventh national census data for the study counties (Figure 1A). A 2021 age distribution map of esophageal cancer incidence identified individuals over 40 years of age as the predominantly affected demographic (Figure 1B), emphasizing the need for age‐targeted screening. Additionally, a population distribution map based on the 2008 regional data was generated (Figure 1C).

An overall population analysis revealed consistent sex distribution patterns, with fewer individuals at age extremes and higher concentrations in the 5–9, 30–34, and 45–54 years age‐groups (Figure 1D, Supporting Information 2 [Figure S3A,B]). The standardized incidence rates (Tables 1, 2, 3) were used to classify the risk: Wudu, Yuzhong, Zhangjiachuan, Lintao, and Jishishan were classified as low‐risk; Minqin, Zhouqu, Dongxiang, and Gaolan as medium‐risk; and Yugu as the only high‐risk region (Figure 1E).

**TABLE 1:** Calculation of standardized esophageal cancer incidence rates for the general population.

Counts	GS_2021_esophagea_number	GS_2021_census_number	Crude rate	Adjust rate	Crude_rate_Per1,000,000	Adjust_Rate_Per1,000,000	LCI	UCI	Risk
Yuzhong	14	473,882	0.000853	0.0000157	2.95	1.57	0.85	3.43	Low
Lintao	19	480,149	0.000843	0.0000209	3.96	2.09	1.25	4.38	Low
Zhangjiachuan	11	244,406	0.00118	0.0000379	4.5	3.79	1.84	8.13	Low
Jishishan	18	239,390	0.00299	0.0000484	7.52	4.84	2.78	8.97	Low
Wudu	38	546,616	0.00137	0.0000485	6.95	4.85	3.41	7.16	Low
Dongxiang	21	290,034	0.00372	0.0000614	7.24	6.14	3.6	10.33	Mediate
Zhouqu	20	125,367	0.00494	0.000113	15.95	11.26	6.38	19.91	Mediate
Gaolan	28	125,157	0.00507	0.000122	22.37	12.19	7.6	20.51	Mediate
Minqin	65	178,470	0.00878	0.000141	36.42	14.08	9.93	21.7	Mediate
Yugu	15	27,762	0.0189	0.000271	54.03	27.09	14.95	64.95	High

*Note:* Counts: counties, representing the names of counties in Gansu Province, China; Group: Ethnic minorities in China; Gs_2021_esophagea_number: Number of esophageal cancer patients in 2021 of Gansu Province; Gs_2021_census_number: Population in the 2021 census of Gansu Province; Crude rate: Unstandardized incidence rate of esophageal cancer; Adjust rate: Age‐standardized incidence rate of esophageal cancer after adjustment; Crude_rate_Per1,000,000: Unstandardized incidence rate of esophageal cancer per 10,000 population; Adjust_Rate_Per1,000,000: Age‐standardized incidence rate of esophageal cancer per 100,000 population; Lcl, lower confidence interval; Ucl, upper confidence interval; Risk: Stratifcation of esophageal cancer risk in regions; high: esophageal cancer ASR > 50/100,000; mediate: esophageal cancer ASR 5–15/100,000; low: esophageal cance ASR < 5/100,000.

**TABLE 2:** Calculation of standardized esophageal cancer incidence rates in the female population.

Counts	GS_2021_esophagea_number	GS_2021_census_number	Crude rate	Adjust rate	Crude_rate_Per1,000,000	Adjust_Rate_Per1,000,000	LCI	UCI
Dongxiang	3	145,606	0.000777062	0.000018	2.06	1.8	0.34	7.54
Jishishan	3	118,533	0.0009341	0.0000162	2.53	1.62	0.31	8.61
Lintao	4	238,013	0.000383847	0.00000845	1.68	0.85	0.23	5.34
Minqin	23	87,161	0.006742598	0.000118144	26.39	11.81	5.5	28.13
Wudu	3	269,643	0.000172685	0.00000739	1.11	0.74	0.15	3.91
Yuzhong	2	230,810	0.000310534	0.00000421	0.87	0.42	0.05	4.24
Zhangjiachuan	3	123,627	0.000672627	0.0000233	2.43	2.33	0.44	10.19
Zhouqu	2	61,973	0.001644191	0.00000848	3.23	0.85	0.09	13.19
Gaolan	4	57,030	0.002319386	0.000023	7.01	2.3	0.58	18.18
Yugu	10	13,239	0.027319808	0.000324505	75.53	32.45	14.99	110.33

*Note:* Counts: counties, representing the names of counties in Gansu Province, China; Group: Ethnic minorities in China; Gs_2021_esophagea_number: Number of esophageal cancer patients in 2021 of Gansu Province; Gs_2021_census_number: Population in the 2021 census of Gansu Province; Crude rate: Unstandardized incidence rate of esophageal cancer; Adjust rate: Age‐standardized incidence rate of esophageal cancer after adjustment; Crude_rate_Per1,000,000: Unstandardized incidence rate of esophageal cancer per 10,000 population; Adjust_Rate_Per1,000,000: Age‐standardized incidence rate of esophageal cancer per 100,000 population; Lcl, lower confidence interval; Ucl, upper confidence interval; Risk: Stratifcation of esophageal cancer risk in regions; high: esophageal cancer ASR > 50/100,000; mediate: esophageal cancer ASR 5–15/100,000; low: esophageal cance ASR < 5/100,000.

**TABLE 3:** Calculation of standardized esophageal cancer incidence rates in the male population.

Counts	GS_2021_esophagea_number	GS_2021_census_number	Crude rate	Adjust rate	Crude_rate_Per1,000,000	Adjust_Rate_Per1,000,000	LCI	UCI
Dongxiang	18	144,428	0.00683	0.000103	12.46	10.34	5.76	18.11
Jishishan	15	120,857	0.00517	0.0000778	12.41	7.78	4.22	15.32
Lintao	15	242,136	0.0013	0.000032	6.19	3.2	1.79	7.49
Minqin	42	91,309	0.0107	0.000162	46	16.16	11.52	28.11
Wudu	35	276,973	0.00248	0.0000836	12.64	8.36	5.79	12.61
Yuzhong	12	243,072	0.00138	0.0000261	4.94	2.61	1.33	5.95
Zhangjiachuan	8	120,779	0.00168	0.0000511	6.62	5.11	2.15	13.01
Zhouqu	18	63,394	0.00834	0.000205	28.39	20.54	11.54	37.13
Gaolan	24	68,127	0.00772	0.000204	35.23	20.42	12.46	35.27
Yugu	5	14,523	0.00756	0.000201	34.43	20.07	6.37	89.57

*Note:* Counts: counties, representing the names of counties in Gansu Province, China; Group: Ethnic minorities in China; Gs_2021_esophagea_number: Number of esophageal cancer patients in 2021 of Gansu Province; Gs_2021_census_number: Population in the 2021 census of Gansu Province; Crude rate: Unstandardized incidence rate of esophageal cancer; Adjust rate: Age‐standardized incidence rate of esophageal cancer after adjustment; Crude_rate_Per1,000,000: Unstandardized incidence rate of esophageal_cancer_per 10,000 population; Adiust Rate Per1,000,000: Age‐standardized incidence rate of esophageal cancer per 100,000 population; Lcl, lower confidence interval; Ucl, upper confidence interval; Risk: Stratifcation of esophageal cancer risk in regions, high; esophageal cancer ASR > 50/100,000; mediate: esophageal cancer ASR 5–15/100,000; low: esophageal cance ASR < 5/100,000.

The geographical analysis showed a lower incidence in the southern versus northern regions, with distinct sex disparities (Figure 1E, Supporting Information 2 [Figure S3C,D]). Men had higher incidence rates in Wudu, Minqin, Zhouqu, Dongxiang, and Gaolan, whereas women had lower rates in these counties along with the Yuzhong, Zhangjiachuan, Lintao, and Jishishan regions (Figure 1F). Overall, a higher incidence was observed in men, except Yugu where women had higher rates. A comparison of cancer case proportions with the general population (Figure 1G) validated the classifications: low‐risk areas had larger general populations, whereas high‐risk areas showed elevated cancer case proportions.

### 4.3 Factors Associated With High Esophageal Cancer Risk

**FIGURE 2:**
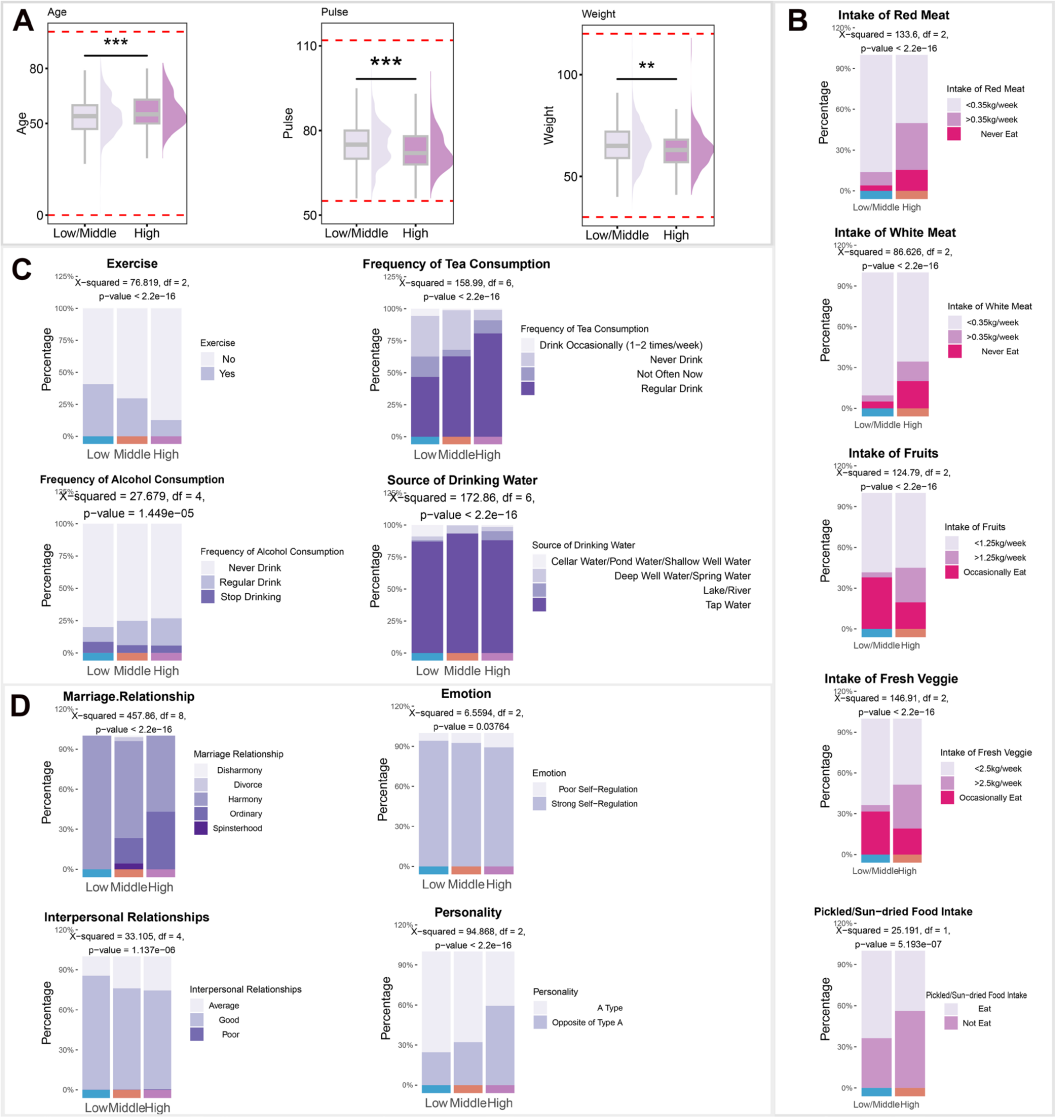
(A) Box plot showing age, pulse rate, weight differences between high‐risk and other esophageal cancer risk areas by the *t*‐test (*n* = 2129). (B) Box plot showing the differences of red meat intake, white meat intake, fruit intake, fresh vegetable intake, and pickled/sun‐dried food intake differences between high‐risk and other esophageal cancer risk areas by the chi‐square test (*n* = 1236). (C) Percentage chart displaying proportional distribution of exercise participation, tea drinking frequency, alcohol consumption, and drinking water sources across esophageal cancer risk levels by the chi‐square test (*n* = 2129). (D) Percentage chart illustrating proportional distribution of marriage relationship status, emotional regulation, interpersonal relationship quality, and personality types across esophageal cancer risk levels by the chi‐square test (*n* = 2129) (***p < 0.001, **p < 0.01).

To identify the risk factors, a food intake survey based on the “2016 Dietary Guidelines for Chinese Residents” and symptom data were collected. High‐risk area individuals showed distinct characteristics: older age at diagnosis, lower pulse rate, and reduced body weight (Figure 2A), suggesting age‐related risk escalation, with slower pulse and weight loss potentially linked to tumor effects, malnutrition, and metabolic changes. No significant sex differences in high‐risk traits were observed (Supporting Information 2 [Figure S4A]).

Diet strongly influenced risk: moderate red/white meat intake (< 0.35 kg/week) correlated with low risk, while excessive/insufficient intake was linked to high risk. Moderate fruit (< 1.25 kg/week) and vegetable (< 2.5 kg/week) intake reduced the disease risk (Figure 2B). Notably, pickled/sun‐dried food consumption was associated with lower risk, whereas high‐salt diets increased the risk (Figure 2B, Supporting Information 2 [Figure S4B]). Low ginger/scallion/garlic intake correlated with a lower risk, and frequent consumption was associated with a higher risk. Fast food intake < 2 times/week reduced risk, while excessive/insufficient intake worsened the outcomes. Reduced grilled food intake, avoidance of fried foods, high dietary fat, rapid eating, and high food temperature were linked to higher risk (Supporting Information 2 [Figure S4C–H]).

Clinically, high‐risk patients more frequently exhibited belching, nausea, melena, vomiting, heartburn, anorexia, dysphagia, odynophagia, chest pain, and early satiety (*p* < 0.05). Acid reflux, back pain, and cervical pain were more common in high‐risk groups, but the results were not statistically significant (Table 4), emphasizing the need for awareness and early screening for persistent symptoms to prevent progression.

**TABLE 4:** Differences in symptoms across esophageal cancer risk regions.

	Influence factor	Regional classification of esophageal cancer risk	Subgroup		Sum	Chi‐squared	*p*
Symptoms	Belching		Yes	No	2063	6.95	8.37e−03
		Low/Middle	6%	94%	1851		3.50e−312
		High	11%	89%	212		4.14e−30
	Back pain		Yes	No	2065	0.89	3.50E−01
		Low/Middle	15%	85%	1853		1.35e−194
		High	18%	82%	212		9.59e−21
	Burping		Yes	No	2062	0.08	7.80E−01
		Low/Middle	15%	85%	1850		8.17e−196
		High	16%	84%	212		4.60e−23
	Nausea		Yes	No	2062	7.01	8.12e−03
		Low/Middle	8%	92%	1850		1.90e−292
		High	13%	87%	212		1.96e−27
	Acid reflux		Yes	No	2064	0.85	3.60E−01
		Low/Middle	13%	87%	1852		3.44e−219
		High	16%	84%	212		1.16e−23
	Abdominal bloating		Yes	No	2065	1.02e−03	9.70E−01
		Low/Middle	16%	84%	1853		3.36e−192
		High	16%	84%	212		1.16e−23
	Black stool		Yes	No	2061	21.81	3.01e−06
		Low/Middle	1%	99%	1849		0.00E+00
		High	6%	94%	212		2.28e−37
	Neck pain		Yes	No	2064	2.72	1.00E−01
		Low/Middle	8%	92%	1852		4.14e−30
		High	11%	89%	212		2.14e−291
	Vomitting		Yes	No	2064	6.04	1.00E−02
		Low/Middle	4%	96%	1852		0.00E+00
		High	8%	92%	212		2.28e−34
	Anemia		Yes	No	1985	0.57	4.50E−01
		Low/Middle	2%	98%	1778		0.00E+00
		High	2%	98%	207		1.13e−42
	Heartburn		Yes	No	2062	7.26	7.04e−03
		Low/Middle	7%	93%	1850		2.19e−298
		High	12%	88%	212		4.32e−28
	Loss of appetite		Yes	No	2065	1.34	2.50E−01
		Low/Middle	13%	87%	1853		7.96e−226
		High	16%	84%	212		1.16e−23
	Weight loss		Yes	No	2054	7.05	7.95e−03
		Low/Middle	8%	92%	1847		1.79e−284
		High	14%	86%	207		9.09e−26
	Difficulty swallowing		Yes	No	2062	24.23	8.55e−07
		Low/Middle	1%	99%	1850		0.00E+00
		High	5%	95%	212		1.05e−39
	Painful swallowing		Yes	No	2065	19.55	9.80e−06
		Low/Middle	1%	99%	1853		0.00E+00
		High	4%	96%	212		1.68e−40
	Chest pain		Yes	No	2064	13.55	2.32e−04
		Low/Middle	2%	98%	1852		0.00E+00
		High	7%	93%	212		1.32e−36
	Early satiety		Yes	No	2064	5.08	2.00E−02
		Low/Middle	4%	96%	1852		0.00E+00
		High	8%	92%	212		4.17e−35

*Note:* Subgroup: Proportion of each option group in the survey questionnaire; Sum: Total number of individuals included in this group of the survey.

### 4.4 Impact of Daily Habits and Psychoemotional Health on Esophageal Cancer Risk

Smoking‐related factors strongly correlated with the esophageal cancer risk: passive smoking duration, smoking history, and cigarette quantity all increased risk (Supporting Information 2 [Figure S4J,K]). In contrast, physical exercise acted as a protective factor, with the proportion of patients who exercised declining as the risk escalated. Regular tea consumption was linked to higher risk, while alcohol intake showed a progressive association with increased risk, and abstinence correlated with lower risk. Notably, patients relying on lake/river water (susceptible to pollution) were predominantly in high‐risk areas, highlighting the need for water purification measures (Figure 2C).

Psychoemotional factors also played key roles. Patients with harmonious marriages were primarily at a low risk, with stable marital proportions decreasing with increasing risk. Weak emotional self‐regulation correlated with higher risk, and strong interpersonal skills were less prevalent in high‐risk groups. Type A personalities (ambitious, confident) revealed low risk, while other personality types demonstrated high risk (Figure 2D). These findings indicate that harmonious marriages, effective interpersonal relationships, robust emotional regulation, and adaptive traits may serve as protective factors, emphasizing the importance of fostering positive familial and social environments to enhance disease resilience.

### 4.5 Construction of a Primary Prevention Risk Model for Esophageal Cancer

**FIGURE 3:**
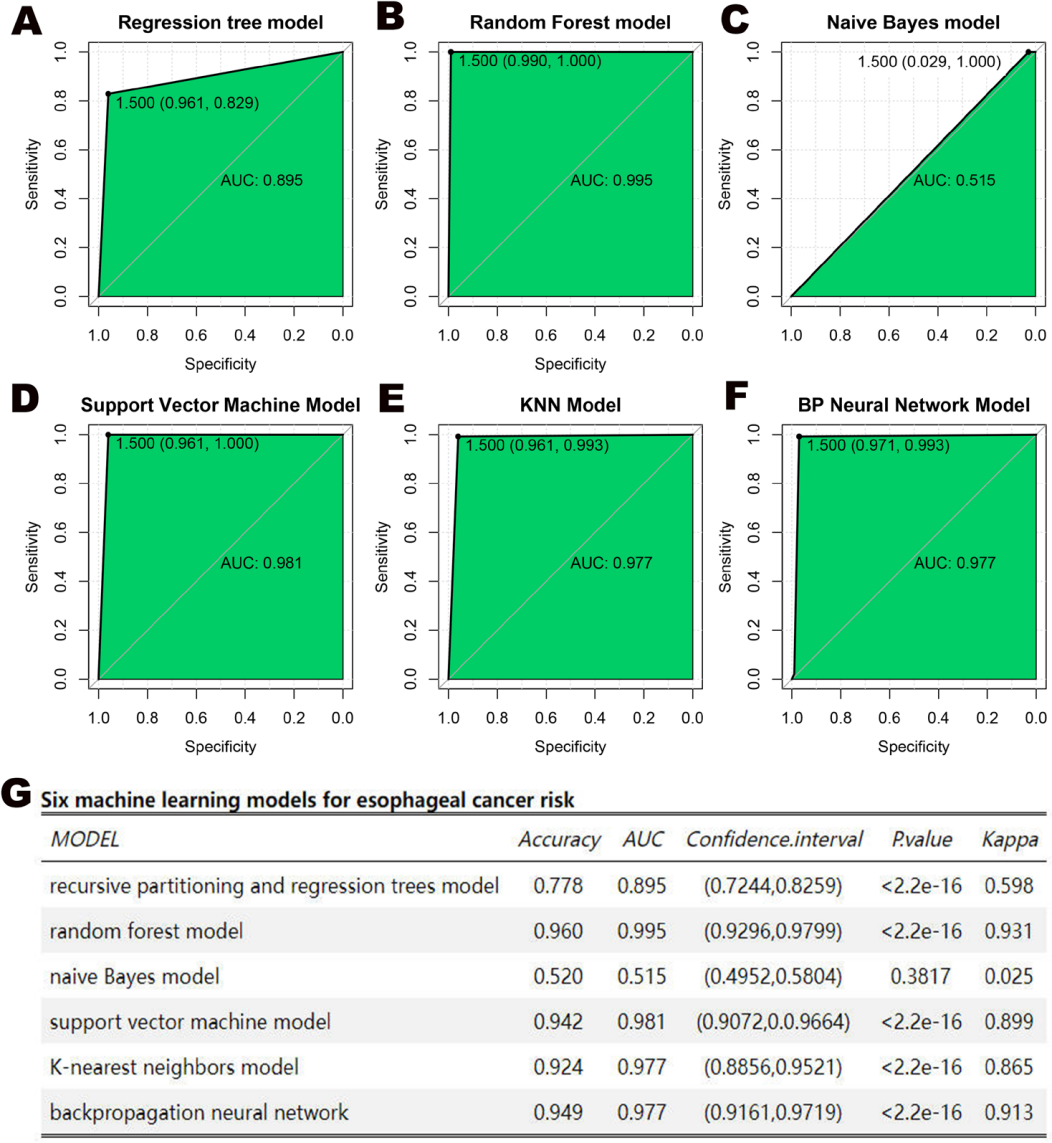
(A–F) The figure shows the area under the ROC curve and the test performance in different model test queues. (G) The figure illustrates the comparison and differences in parameter performance among six different models.

To enhance esophageal cancer risk assessment, 64 indicators (demographics, lifestyle, diet, psychology, symptoms, comorbidities) were compiled to develop primary prevention models using artificial intelligence. Six algorithms were employed: recursive partitioning/regression trees, random forest, naive Bayes, support vector machine, K‐nearest neighbors, and backpropagation neural network. Validation cohort assessment showed that the random forest model had superior performance with an AUC of 0.995 and accuracy of 0.960, outperforming others (AUC: 0.895–0.981; accuracy: 0.520–0.949; Figure 3A–G).

The random forest analysis identified 15 key risk factors, including gastric cancer risk grading, back pain, and daily sugar intake. Correlation analysis and evaluation of prediction accuracy reduction/Gini coefficient refined the focus to 8 crucial indicators, with gastric cancer risk grading and daily sugar intake prominent. An interaction effect analysis showed that these two factors had the smallest average minimum depth, indicating the strongest interaction (Supporting Information 2 [Figure S5A–D]). Individuals at high risk of esophageal cancer were predominantly in the extremely high gastric cancer risk category; moderate‐risk individuals were in both extremely high and high gastric cancer risk categories, while low‐risk individuals were mainly in the moderate‐to‐high gastric cancer risk categories. Increased moderate daily sugar intake correlated with higher esophageal cancer risk, with a notable decline in those with optimal intake (Supporting Information 2 [Figure S5E,F]).

### 4.6 RHBDF2 Suppresses Esophageal Tumor Progression via EMT and KRAS Signaling

**FIGURE 4:**
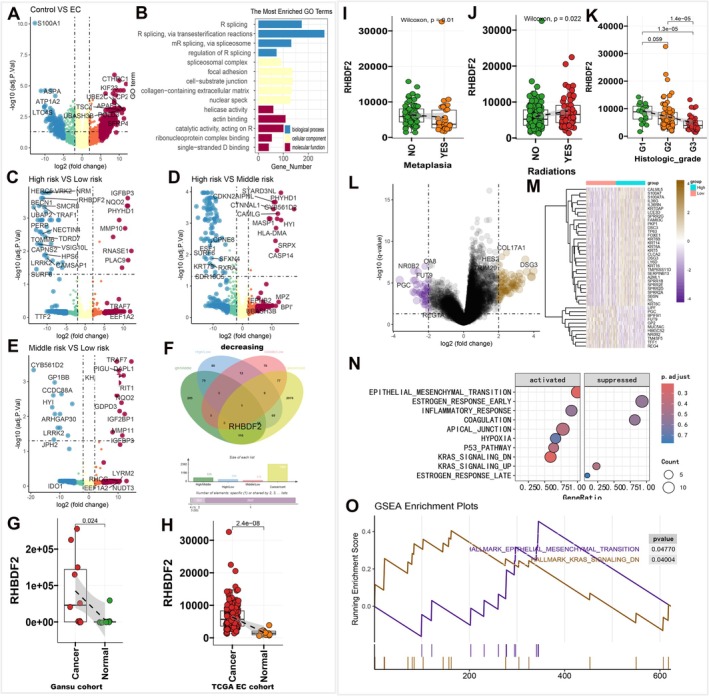
(A) Volcano plot showing differentially expressed genes (DEGs) between esophageal tumor and normal tissues. (B) Bar plots showing biological pathways enriched by DEGs in esophageal cancer versus control samples. (C–E) Volcano plots showing DEGs from pairwise risk group comparisons: high‐risk versus medium‐risk (C), high‐risk versus low‐risk (D), and medium‐risk vs. low‐risk (E). (F) Venn diagram of lowly expressed genes across esophageal cancer risk groups. (G, H) Boxplots showing RHBDF2 expression differences between tumor and control tissues in the Gansu cohort (G, *n* = 20) and TCGA ESCA cohort (H, *n* = 198) by the *t*‐test. (I–K) Boxplots showing RHBDF2 expression differences across tissue metaplasia status, radiotherapy status, tumor grades by the Wilcoxon rank‐sum test (*n* = 162). (L–M) Volcano plot and heatmap showing differentially expressed genes (DEGs) between RHBDF2‐high and RHBDF2‐low expression groups in esophageal cancer. (N) Bubble plot showing Hallmark pathways regulated by DEGs, identified via Gene Set Enrichment Analysis (GSEA). (O) GSEA enrichment plots demonstrating significant pathway enrichment between RHBDF2‐high and RHBDF2‐low expression groups in the TCGA‐ESCA cohort.

High‐throughput proteomic sequencing identified 2392 functionally annotated DEGs between patients with esophageal cancer and controls (Figure 4A), with GO enrichment analyzing their biological functions (Figure 4B). Pairwise DEG analyses among high‐, medium‐, and low‐risk groups (Figure 4C–E) identified 179 upregulated/535 downregulated (high vs. medium), 112 upregulated/294 downregulated (high vs. low), and 220 upregulated/175 downregulated (medium vs. low) genes. Venn analysis revealed RHBDF2 (decreasing in cancer high risk grade), highly expressed in esophageal cancer of Gansu's cohort and TCGA‐ESCA cohort (Figure 4F–H); RHBDF2 was focused on owing to limited prior reports.

RHBDF2 expression varied by country but not ethnicity, was lower in Barrett's esophagus, higher in squamous cell carcinoma than adenocarcinoma, but showed no age/sex differences (Supporting Information 2 [Figure S6A–F]). RHBDF2 expression was lower in metaplasia, increased post‐radiotherapy, and decreased with advancing pathological stage (consistent with regional risk trends) (Figure 4I–K).

To investigate the underlying mechanisms of the above expression characteristics of RHBDF2, we analyzed immune infiltration in patients with esophageal cancer. Notably, no significant differences in immune cell levels were observed between the high and low‐RHBDF2 expression groups (Supporting Information 2 [Figure S6G]). GSEA of DEGs between these groups revealed RHBDF2 activates epithelial mesenchymal transition and KRAS signaling down pathways (Figure S6L,Q, Supporting Information 2 [Figure S6A]). KEGG enrichment showed activation of cytokine‐cytokine receptor interaction, neuroactive ligand‐receptor interaction, and pathways in cancer pathways, but inhibition of hedgehog signaling. A transcription factor analysis identified RHBDF2‐activated FOSL1, JUND, and FOS (Supporting Information 2 [Figure S6B–E]). This is the first study showing that RHBDF2 downregulation correlates negatively with pathological staging, suppressing tumor progression via EMT/KRAS signaling, serving as a potential diagnostic biomarker and therapeutic target.

## 5 Discussion

This 9‐year comprehensive study of the epidemiology of esophageal cancer in 10 districts and counties in Gansu Province highlights significant regional differences in the incidence trends and underscores the importance of addressing health inequalities. At the same time, the potential influence index of northwest China on patients with esophageal cancer was studied, and it was regarded as an important significance for the comprehensive evaluation of individual cancer risk. Notably, Yugur County emerges as a high‐risk area, which is predominantly inhabited by the Yugur ethnic group. Research indicates a marked divergence in the gut microbiome between the Yugur and Han populations, which can be attributed to the region's unique geographical and cultural characteristics as well as the traditional dietary practices and lifestyle choices of the Yugur people [15, 16]. Furthermore, Wang et al. suggest that variations in the awareness of esophageal cancer, along with factors such as occupation and educational levels, may contribute to the heightened incidence observed in this demographic [17]. Women from Yugur County have atypically high esophageal cancer incidence, requiring sex‐specific factor exploration and targeted interventions. Those > 40 years constitute the primary affected population and should be prioritized for screening; standardized incidence‐based risk zoning aids public health prioritization.

In examining the dietary patterns, our investigation reveals that a moderate intake, rather than excessive consumption, of various food types is crucial, with a notable association between reduced risk and a weekly intake of red and white meat below 0.35 kg. Such moderation may contribute to weight management and a lower body mass index, subsequently reducing the incidence of esophageal cancer [18, 19, 20]. Additionally, the intake of fruits and vegetables demonstrates an inverse correlation with risk; moderate consumption—less than 1.25 kg per week for fruits and 2.5 kg for vegetables—effectively mitigates risk. Diets rich in fresh fruits and vegetables, which deliver essential nutrients and antioxidants, are linked to a diminished risk of esophageal cancer by protecting cellular health [21]. Furthermore, avoiding processed meats and foods that exacerbate chronic acid reflux, a known risk factor for esophageal adenocarcinoma, is strongly recommended [22]. These findings underscore the significance of maintaining a balanced dietary structure as a preventive measure against esophageal cancer, emphasizing that dietary modifications are integral to primary prevention efforts.

Our study also identifies that the avoidance of moldy, pickled, and sun‐dried foods containing nitrosamines as well as barbecue foods high in polycyclic aromatic hydrocarbons may provide protective benefits against esophageal cancer, as supported by existing research [10, 23, 24]. However, in high‐risk areas, there is a notable tendency to avoid fast food and convenience options, potentially attributed to a lack of overall nutritional resources, which may result in compensatory consumption of high‐calorie foods [16, 25]. Similarly, the use of spices such as ginger, scallions, and garlic showcases complex dietary strategies; these ingredients exhibit protective effects owing to their antioxidant and anti‐inflammatory properties. Notably, curcumin in ginger possesses antitumor characteristics relevant to various cancers, including esophageal, lung, and colorectal cancers [26]. A significant body of evidence suggests that garlic may lower the risk of esophageal cancer, possibly owing to the antitumor properties of its diallyl disulfide content [27, 28]. However, an excessive intake of these ingredients can increase risk, thus highlighting the necessity of advocating moderation in seasoning use. Consequently, public health initiatives focusing on these lifestyle modifications will be paramount in decreasing the incidence of cancer.

Tobacco and alcohol consumption are well‐established as the most significant risk factors for esophageal squamous cell carcinoma, with their synergistic effects exponentially elevating the cancer risk [19, 23, 29]. Meanwhile, tea consumption has emerged as a potential risk factor [30, 31, 32]. In addition, the habit of consuming extremely hot tea or milk tea in high‐altitude regions has been linked to elevated risks, particularly for esophageal squamous cell carcinoma [18, 33, 34, 35, 36].

The overlapping symptoms of dyspepsia in esophageal and gastric cancers present challenges in early diagnosis. Our analysis suggests the necessity of distinguishing between functional dyspepsia and organic pathology, emphasizing the critical role of identifying specific symptoms to guide targeted investigations and interventions for cancer [37, 38].

The quality of marital relationships and interpersonal skills has been correlated with a reduced risk of esophageal cancer. Establishing supportive social networks and nurturing positive family dynamics contribute significantly to an enhanced quality of life and diminished cancer risk. We propose incorporating psychological support and counseling into primary prevention strategies as essential components in reducing the esophageal cancer risk and improving public health outcomes.

Furthermore, we advocate for the development of a first‐level prevention risk model for esophageal cancer utilizing artificial intelligence and deep learning methodologies. By synthesizing diverse factors, including dietary habits, lifestyle behaviors, and psychological well‐being, this model provides a robust scientific foundation for risk assessment, aiding individuals in understanding their health risks and implementing effective preventive strategies. The efficacy and accuracy of the random forest model present a practical approach to facilitating esophageal cancer screening and prevention in everyday life. Notably, the interplay between gastric cancer risk and sugar intake emerges as a critical factor influencing the esophageal cancer risk; limiting sugar consumption may mitigate the risk of Barrett's esophagus and subsequent esophageal cancer [39], although other studies highlight the potential for sugar metabolism pathways to drive genomic changes conducive to cancer proliferation [40]. Research indicates that sugar and fat intake significantly impact the manifestation and course of chronic gastrointestinal diseases [41], aligning with our findings that underscore the protective role of moderate dietary intake against esophageal cancer.

The study found that RHBDF2 exhibits high expression levels in esophageal cancer, and its expression level exhibits a negative correlation with pathological staging, suggesting that it may serve as an important prognostic biomarker. Functionally, RHBDF2 significantly suppresses the progression of esophageal cancer by inhibiting the epithelial‐mesenchymal transition (EMT) and KRAS signaling pathways. This finding is supported by multi‐omics analyses: both transcriptomic and proteomic data show that low expression of RHBDF2 leads to the activation of the EMT and KRAS signaling pathways, which in turn promotes tumor invasion and metastasis [42, 43].

The innovation of this study was combining epidemiological surveys, clinical sample sequencing (identifying 2392 differentially expressed genes) and TCGA data analysis, which ensures the reliability of the results. Through GSEA and Kyoto Encyclopedia of Genes and Genomes enrichment analyses, the core pathways regulated by RHBDF2 (EMT and KRAS) were clarified, and its association with processes such as cytokine‐cytokine receptor interaction and inhibition of Hedgehog signaling was identified. The expression characteristics of RHBDF2 (e.g., upregulation after radiotherapy, differential expression between adenocarcinoma and squamous cell carcinoma) provide a basis for its use as a therapeutic target. For example, radiotherapy may enhance tumor‐suppressive effects by upregulating RHBDF2.

Furthermore, the study also constructed a machine learning‐based risk prediction model (random forest model with AUC = 0.995), providing a practical tool for the early screening of esophageal cancer. Future work needs to further validate the function of RHBDF2 in vitro and in vivo, and explore whether pathways targeting RHBDF2 can provide new strategies for esophageal cancer treatment.

## Author Contributions

**Duojie Zhu:** conceptualization (equal), data curation (equal), methodology (equal), project administration (equal), resources (equal), validation (equal), visualization (equal), writing – original draft (equal), writing – review and editing (equal). **Yinggang Che:** conceptualization (equal), formal analysis (equal), software (equal), validation (equal), visualization (equal), writing – original draft (supporting). **Huijuan Cheng:** conceptualization (equal), data curation (equal), investigation (equal), methodology (equal), resources (equal), supervision (equal). **Weiqi Wang:** conceptualization (equal), formal analysis (equal), project administration (equal), resources (equal), validation (equal), writing – review and editing (equal). **Yumin Li:** conceptualization (equal), data curation (equal), funding acquisition (equal), methodology (equal), project administration (equal), resources (equal), software (equal), supervision (equal), validation (equal), writing – original draft (equal), writing – review and editing (equal).

## Funding

This work was supported by Major Science and Technology Project of Gansu Province (20ZD7FA003, 22JR9KA00, 22ZD6FA050), the Second Hospital of Lanzhou University Cuying Science and Technology Innovation Plan Project (CY2021‐BJ‐A19), the Shaanxi Province key research and development plan (2023‐YBSF‐230).

## Ethics Statement

This study was conducted in accordance with the Declaration of Helsinki and was approved by the Ethics Committee of Lanzhou University Second Hospital (Approval No. [2024A‐059]).

## Consent

Written informed consent was obtained from all participants prior to their involvement in the study.

## Conflicts of Interest

The authors declare no conflicts of interest.

## Supporting information


**Data S1:** cam471605‐sup‐0001‐Supinfo1.pdf.

**Data S2:** cam471605‐sup‐0002‐Supinfo2.pdf.

## Data Availability

The datasets and code that support the findings of this study are available from the corresponding author upon reasonable request.
