# Supplementary material for: Epidemiology of Risk Stratification, Machine Learning Early Prediction Model, and Tumor Suppressive Mechanism of RHBDF2 in Esophageal Cancer in Gansu Province
Source: Cancer Med. 2026 Mar 17;15(3):e71605. doi: 10.1002/cam4.71605 (PMC13140592; doi:10.1002/cam4.71605)
Supplement: Supplementary file 1 — Data S1: cam471605‐sup‐0001‐Supinfo1.pdf. [file CAM4-15-e71605-s002.pdf]

ID number:

Information questionnaire of western environment and high-incidence tumor research project (2022)

Last name: ________________

Sex: ________________

Date of birth: ________________

Age: ________________

Ethnic group: ________________

Home address: ________________

Survey date: ________________

Contact telephone number:_________

Lanzhou University Medical Center Project Executive Office

Informed consent form of western environment and high-incidence tumor research project

This project mainly studies the environmental risk factors related to high-incidence tumors in the west of China, constructs the monitoring points of high-risk groups in high-incidence areas, and takes communities or townships or villages in high-incidence areas as units to establish the observation points of susceptible groups. To study the influence of living habits, mental factors, living conditions, family history, economic situation and social factors on the high incidence of cancer in susceptible population. Establish a cohort of high-risk groups exposed to environmental factors of high-incidence tumors in western China, screen susceptible groups in high-incidence areas, set up a control group, conduct intervention research, and conduct long-term follow-up and follow-up monitoring to study environmental factors of tumor occurrence and key factors of prevention and control. To study the characteristics of high-risk factors of different regions, different environments and different ethnic groups, especially the environmental risk factors of high-risk tumors of ethnic minorities in western China.

Principle of confidentiality

Your relevant information will be kept strictly in accordance with relevant laws and will not be made public, and all your information will be kept confidential. Voluntary principle

Participation in the project is completely voluntary.

Please decide for yourself whether to participate or not. Self-declaration

I have read the informed consent form and understood all the information. Some problems have been discussed with the inspection team and solved satisfactorily.

Signature of participants:____________________date_____________year_____moon____sun

Questionnaire on risk factors of western environment and high-incidence tumor research project

A basic information

A1 What is your education level? ||

1. illiterate	2. primary school	3. Junior high school	4. High school/technical secondary school/technical school

A2

6. universities and colleges	7. undergraduate course	8. Graduate students and above

A3 What is your occupation? ||

1. Personnel of public institutions/civil servants 2. Enterprise personnel/workers 3.self-employed

4. Freelancer	5. Farmers/migrant workers	6. unemployed people
7. Retired personnel	8. other	

A4 What kind of medical insurance do you currently have? ||

1. Medical insurance for urban workers	2. Medical insurance for urban residents	3. New rural cooperative medical system

A5 How many people live together in your family now?__human

A6 Your personal and family income and expenditure:

- A6.1 What was the total income of your family last year? | |

10,000 or less	21,000-29,000	33,000-49,000
45,000-69,000	570,000-100,000	More than 610,000

B Smoking and drinking

B1 Do you smoke (those who smoke more than one cigarette a day for more than 6 months are defined as smoking)? ||

1. No, never (turn B3). 2. Yes, I'm still smoking. 3. Used to smoke, but

now quit smoking B2 What kind of cigarettes do you smoke ||

Step 1: cigarettes 2. Dry tobacco 3. hookah

- B2.1 If you still smoke or have smoked, how many cigarettes do you smoke on average every day (1 two leaves =50 cigarettes)? ||____| branch
- B2.2 If you still smoke or have smoked, how many years have you smoked

after deducting the years of quitting smoking? |||| B3 Did you smoke passively (did

anyone smoke frequently in the environment where you lived and/or worked for a long

time)? | |

1. be Step 2: no

- B3.1 If yes, how many years of passive

smoking? |||| B4 Are you exposed to biofuels such

as wheat straw and coal? | |

1. be Step 2: no

B5 Do you often drink alcohol (usually at least once a week for more than 6 months)? ||

1. No, never drink Step 2 drink regularly 3. Quit drinking

- B5.1 types of drinking

type	Yes/no	How much do you drink every week?	How many years have you been drinking?	
beer	||	||____| Bottle/week	||____| year	
Chinese hard liquor	||	||____| Jin/week	||____| year	Alcohol | _ |
Homemade liquor	||	||____| Jin/week	||____| year	Alcohol | _ |
other	||	||____| Jin/week	||____| year	

- B5.2 drinking together____year
- B5.3 If you have stopped drinking at present, stop drinking._______year

B6 Do you often drink tea (usually at least 3 times a week for more than 6 months)? ||

1. No, never drink. Step 2 drink regularly I used to, but now I don't drink it often. 4. Drink occasionally (1-2 times a week)

- B4.1 Types of tea drinking (1. No 2, Yes)

type Yes/no How much do you drink every month? How many years have you been drinking?

scented tea ||||| Two months ||________________________| year

green tea ||||| Two months ||________________________| year

brick tea ||||| Two months ||________________________| year

black tea |||||| Bi-monthly |||| Year B7 Your drinking water mainly

comes from: ||

1. cellar water, pond water and shallow well water 2. Lake water, river water 3. Deep well water, spring water 4. Tap water

B8 Do you often take part in manual labor (usually more than 3 times a week, each time for more than 30 minutes)? ||

Step 1: no 2. Yes

- B8.1 the way you exercise. (1. No 2, Yes)

type	Yes/no	How many times a week?	How many years have you exercised?
run	||	||| times/week	||____| year
good-bye	||	||| times/week	||____| year
labour	||	||| times/week	||____| year
other	||	||| times/week	||____| year

C eating habits

C1 In the past two years, your personal average intake of the following foods (the personal average can be calculated by dividing the total family by the population)?

- C1.1 Fresh vegetables (excluding potatoes, uncooked raw weight): ||

  Step 0 eat occasionally 1. < 5kg/week 2. ≧5 kg per week

- C1.2 Fresh fruit (raw weight without skin): ||

  Step 0 eat occasionally 1. < 2.5kg/week 2. ≧ 2.5kg/week

- C1.3 Red meat (refers to pigs, cattle and mutton, uncooked raw weight): | |

  0. Never eat 1.<7 Two weeks/week 2. >7 two weeks/week

- C1.4 White meat (chicken, duck, fish, uncooked raw weight):| |

  0. Never eat 1.<7 Two weeks/week 2. >7 two weeks/week

- C1.5 Coarse grains (coarse grains except white flour and rice, uncooked raw weight): | | 0. Never eat. 1. < 1kg/week 2. ≧ 1kg/week

C2 Do you often eat the following foods? 0. Never 1. Rarely (-2 times/week) 2. Often

C2.1 salted food	||	C2.2 fried food	||
C2.3 moldy food	||	C2.4 onion, ginger and garlic	||

C2.5 foreign fast food and convenience food | | C2.6 barbecue food | | C3 Your usual eating habits:

C3.1	Hot and cold degree:	1. Hot drinks and food	Step 2 be moderate	Step 3 be cool	||
C3.2	Dry hardness:	Step 1 dry hard	Step 2 be moderate	Step 3 be soft	||
C3.3	Taste:	1. Heavy salt	Step 2 be moderate	Step 3 be light	||
C3.4	Grease:	1. Higher	Step 2 be moderate	3. Low	||
C3.5	Eating speed:	Step 1 be quick	Step 2 be moderate	Step 3 be slow	||
C3.6	Sugar:	Step 1 eat often	Step 2 be moderate	3. Less	||

D personality and physical problems

question	conte nt	optio n
D1 personality	1. Type A (quick-moving, impatient, aggressive and excitable)<br>2.Contrary to a	||
D2 emotion	1.Strong self-regulation, easy to adapt to the environment<br>2.Poor self-regulation, not easy to adapt to the environment	||
D3 married life	Step 1 be harmonious 2. General 3. disharmony<br>Step 4 rupture 5. Unmarried	||
D4 interpersonal relationship	1. Good 2. General 3. Poor	||
D5 Has there been any major mental trauma in recent years?	1 Yes and 2 No.	||
Does D6 often sulk?	1 Yes and 2 No.	||
D7 mental state	Are you in a state of mental depression for a long time?<br>1. Yes 2. No (if not, skip D8)	||
D8 causes of depression	1. Frustrated at work. 2. Death of relatives. 3. Family disharmony.<br>4. Accidents5. Others	||

E history of upper digestive tract

Disease name	Yes/No (1= Yes, 2= No)	Age at diagnosis
E1 Gastric and duodenal ulcer	||	||____| years old
E2 reflux esophagitis	||	||____| years old
E3 gastroenteritis	||	||____| years old
E4 hepatitis	||	||____| years old
E5 cirrhosis	||	||____| years old
E6 anemia	||	||____| years old
E7 tumor	||	||____| years old
E8 Others (please specify):		||____| years old

F family history

F1 Does anyone in your family have a tumor? ||	1. Yes 2. No.
F2 family history	

family ties	Tumor name	number of people
||_____|__|	||_____|___|___|	||

||____|__|	||____|__|__|	||
||____|__|	||____|__|__|	||
||____|__|	||____|__|__|	||

G have you had the following symptoms in the past six months or a year? (1. Yes, 2 No)

symptom	Yes/ no	If yes, state the time and frequency.
G1 dysphagia	||	
G2 swallowing pain	||	
G3 retrosternal pain	||	
G4 back pain	||	
G5 retrosternal cautery	||	
G6 abdominal distension	||	
G7 heartburn	||	
G8 acid regurgitation	||	
G9 nausea	||	
G10 vomiting	||	
G11 loss of appetite	||	
G12 early satiety	||	
G13 belching	||	
G14 is easily fatigued	||	
G15 stomachache	||	
G16 black stool	||	
G17 Weight loss	||	

H physical examination

Height cm	Weight kg	Pulse (beats/minute)	Blood pressure mmHg
||____|__|	||____|__|	||____|__|	||____|__|/||____|__|

Name of investigator (signature):____________A u d i t o r ' s n a m e (s i g n a t u r e) :

Survey date: ||__|__|__| Year ||_| Month |||_______________| Date of review: ||____|____|_____| Year ||-| Month |||_______
