# Supplementary material for: Epidemiology of Risk Stratification, Machine Learning Early Prediction Model, and Tumor Suppressive Mechanism of RHBDF2 in Esophageal Cancer in Gansu Province
Source: Cancer Med. 2026 Mar 17;15(3):e71605. doi: 10.1002/cam4.71605 (PMC13140592; doi:10.1002/cam4.71605)

Supplement Material 2

Supplement Figure S1. (A) Distribution of peptide lengths in protein samples. (B) Violin plots illustrating the expression levels of normal and tumor tissue samples. (C) Correlation plots among protein-sequenced samples. (D) PCA scatter plots depicting the distribution of esophageal cancer and normal samples.

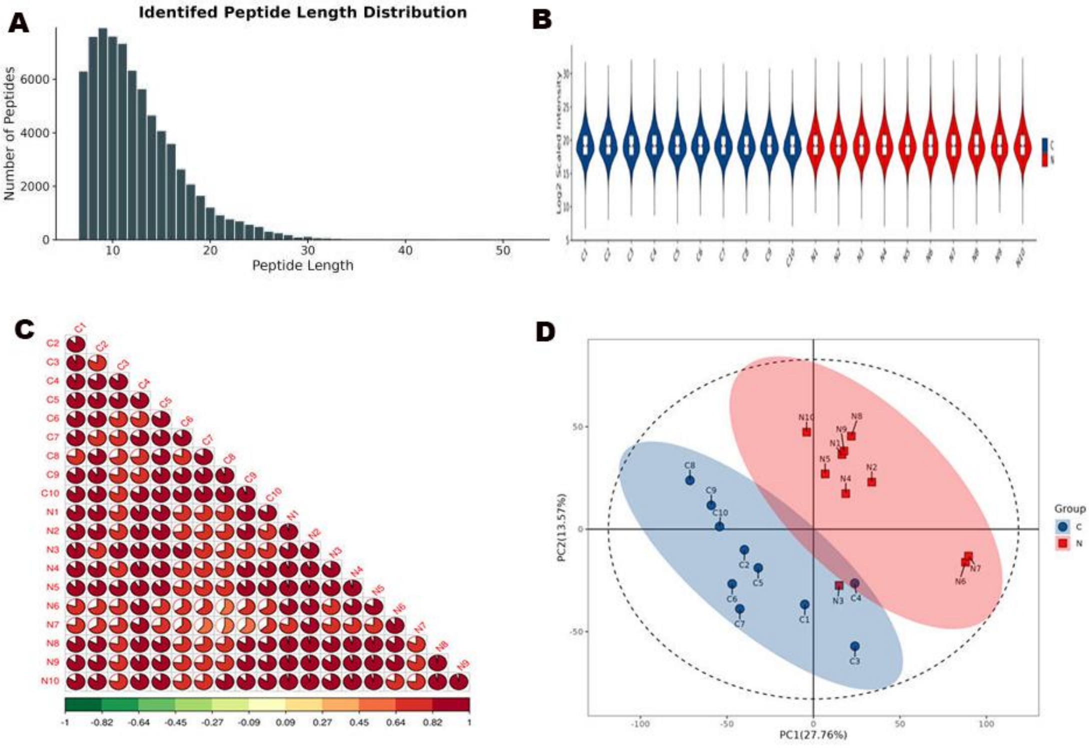

Supplement Figure S2 (A) The line chart shows the changes in the number of esophageal cancer cases in 10 research counties in Gansu Province from 2013 to 2021 over a duration of 9 years. (B) The bar chart displays the total number of esophageal cancer cases in each county of the 10 research counties in Gansu Province from 2013 to 2021 over these 9 years. (C) The bar chart shows the total population from the seventh national census of China in 10 research counties in Gansu Province from 2013 to 2021 over these 9 years. (D) The bar chart illustrates the incidence rate of esophageal cancer in 10 research counties in Gansu Province in 2021. (E) The bar chart presents the prevalence rate of esophageal cancer in 10 research counties in Gansu Province in 2021.

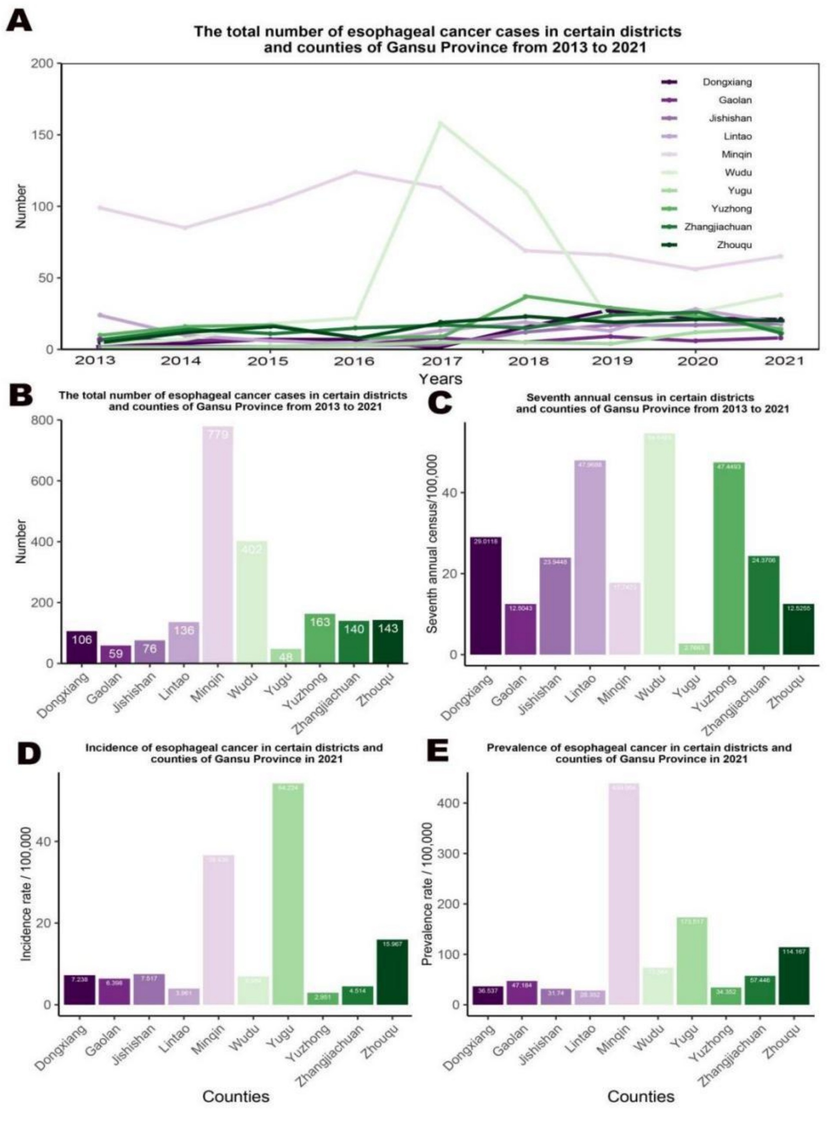

Supplement Figure S3 (A) Stacked bar chart showing female population composition ratios across Gansu study counties based on the 2021 Seventh National Population Census. (B) Stacked bar chart showing the male population composition ratios across Gansu study counties based on the 2021 Seventh National Population Census. (C) A geographic distribution map of standardized esophageal cancer risk levels (female) across Gansu study counties. (D) A geographic distribution map of standardized esophageal cancer risk levels (male) across Gansu study counties.

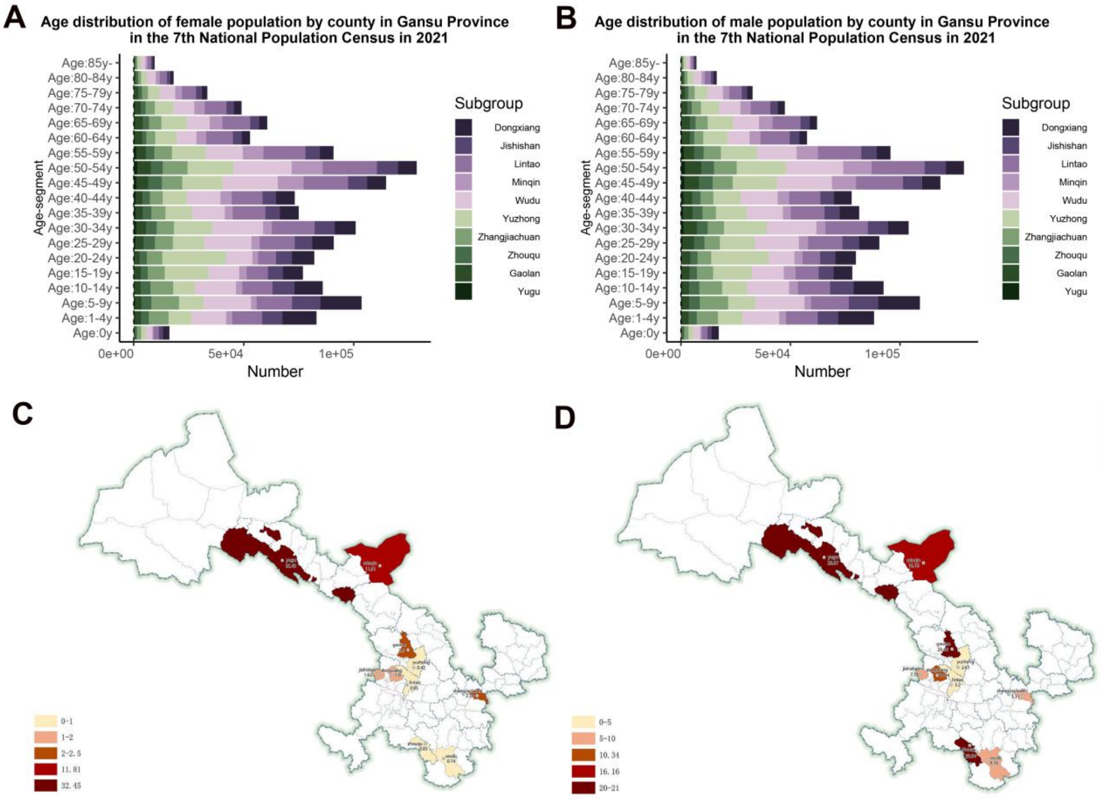

Supplement Figure S4 (A) Box plot showing sex distribution differences between high-risk and other esophageal cancer risk areas by the chi-square test (n=2129). (B-H) Box plot showing the differences of sodium intake in dietary, grilled/barbecued food intake, frequency of fast-food intake, grilled/barbecued food intake, frequency of fried food consumption, fat intake level in diet, pace of eating, temperature preference in eating between high-risk and other esophageal cancer risk areas by the chi-square test (n=1236). (J-K) Box plot showing the differences in years of passive smoking history (n=588), years of smoking (n=494), and the average number of cigarettes smoked (n=509) across esophageal cancer risk levels by the t-test (n=588, 494, 509). (***: P<0.001, **: P<0.01, *: P<0.05, ns: not significant)

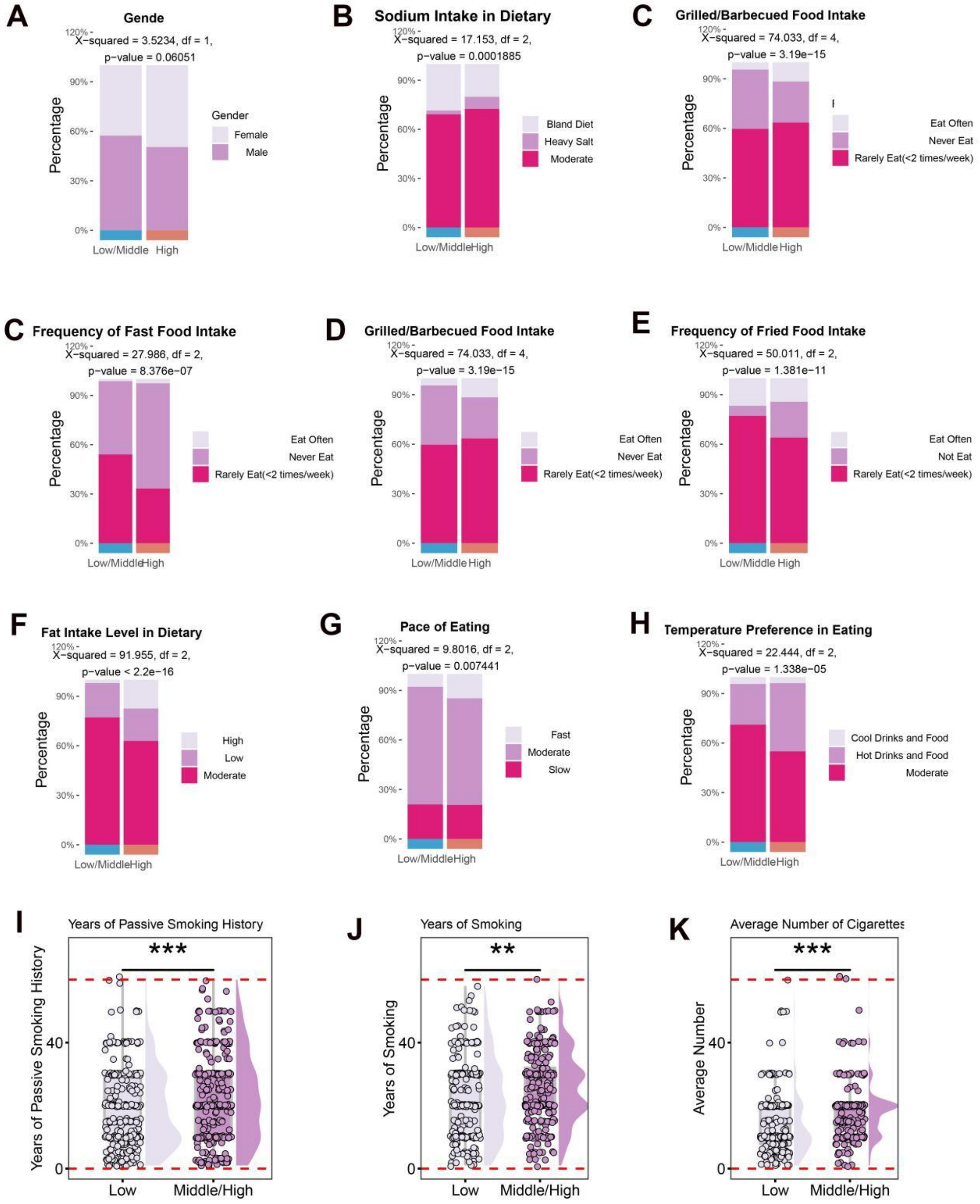

Supplement Figure S5 (A) Chart displaying the top 15 esophageal cancer risk factors based on the minimum depth and mean distribution. (B) Correlation of ranking parameters generated in the random forest model. (C) Top 8 esophageal cancer risk factors ranked by accuracy decrease and Gini decrease parameters. (D) The average minimum depth of pairwise interaction relationships among the top 30 esophageal cancer risk factors. (E) Distribution of individuals across gastric cancer risk strata in different esophageal cancer risk areas. (F) Distribution of individuals by sugar intake frequency across different esophageal cancer risk areas.

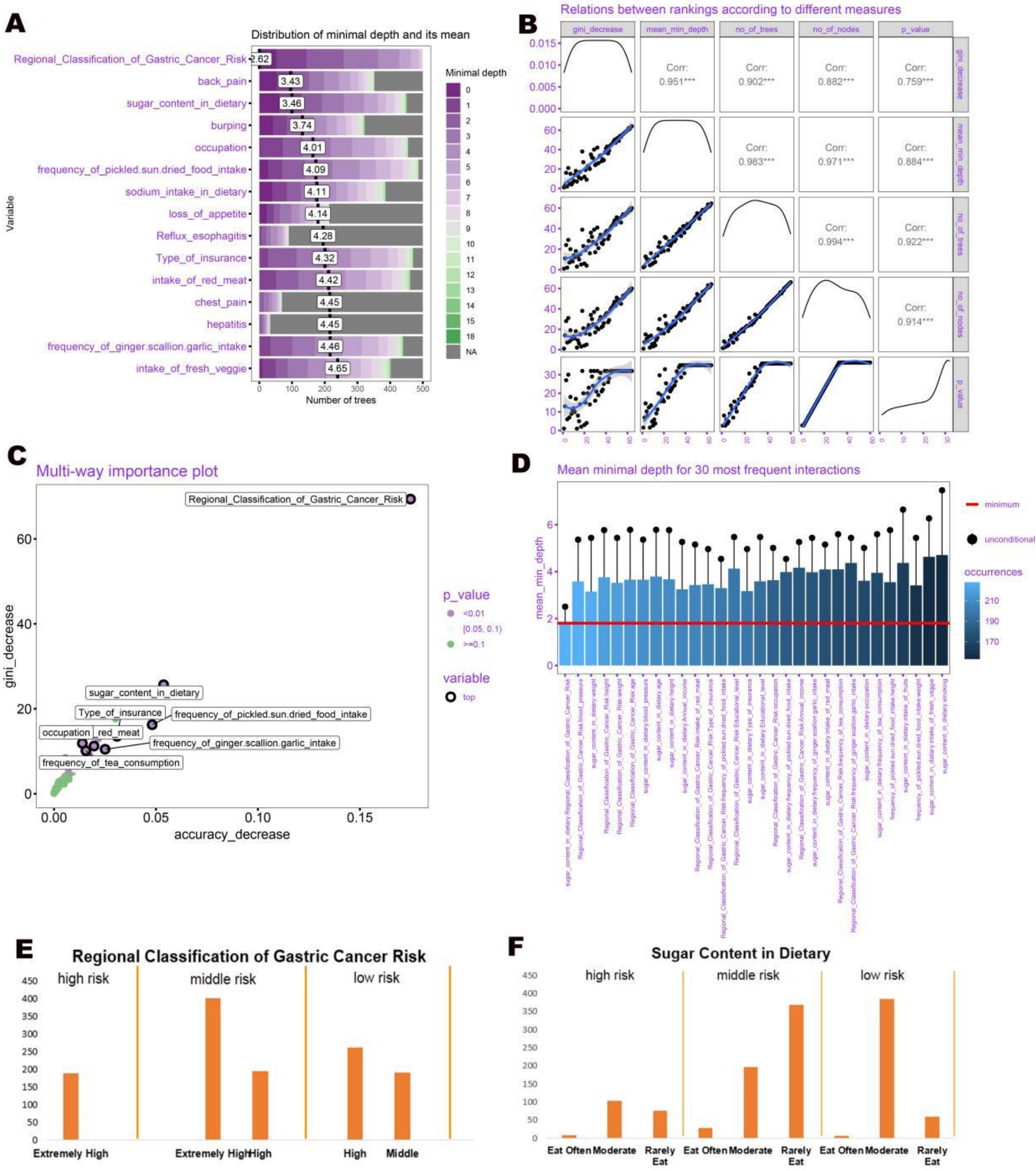

Supplement Figure S6 (A-E) Boxplots showing the RHBDF2 expression differences across countries, ethnic groups, Barrett's esophagus status, tissue types, and sexes (For comparisons between two groups, the Wilcoxon rank-sum test (two-tailed) was used. For comparisons between multiple groups (> three groups), the Kruskal-Wallis test was used, n=162). (F) Boxplot showing age-stratified RHBDF2 expression differences by the t-test (n=162). (G) Boxplot illustrating the differential characteristics of immune cells between RHBDF2-high and RHBDF2-low expression groups by the Wilcoxon rank-sum test (n=185). (*: <0.05, ns: not significant)

.

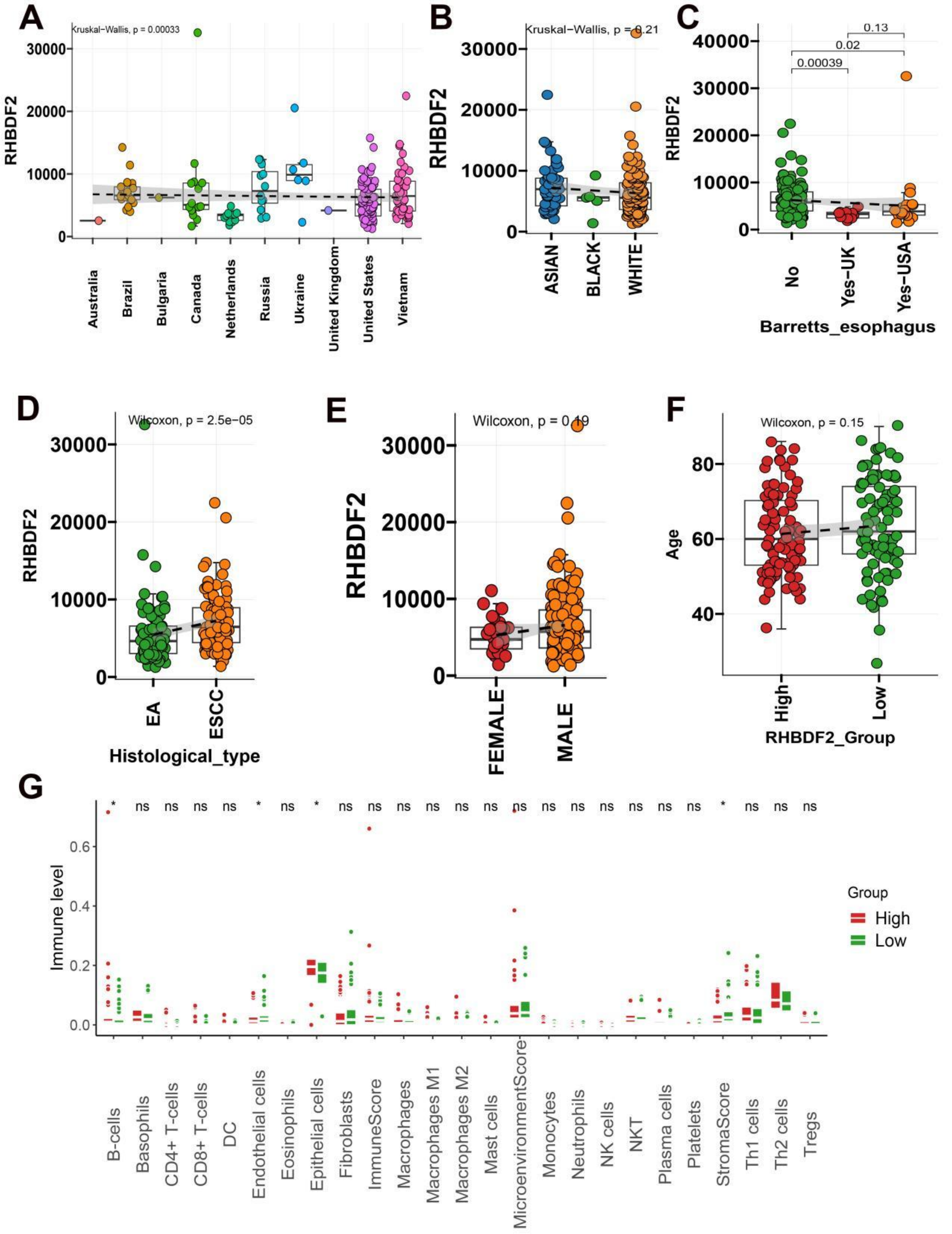

Supplement Figure S7 (A) Gene Set Enrichment Analysis (GSEA) gene-set association visualization plot showing the association between differentially expressed genes and functional gene sets. (B) Bubble plot showing KEGG pathways regulated by DEGs, identified via GSEA. (C) Bubble plot showing transcription factors regulated by DEGs. (D-E) GSEA enrichment plots demonstrating activated and repressed transcriptional regulatory genes within transcription factor target gene sets between RHBDF2-high and RHBDF2-low expression groups.

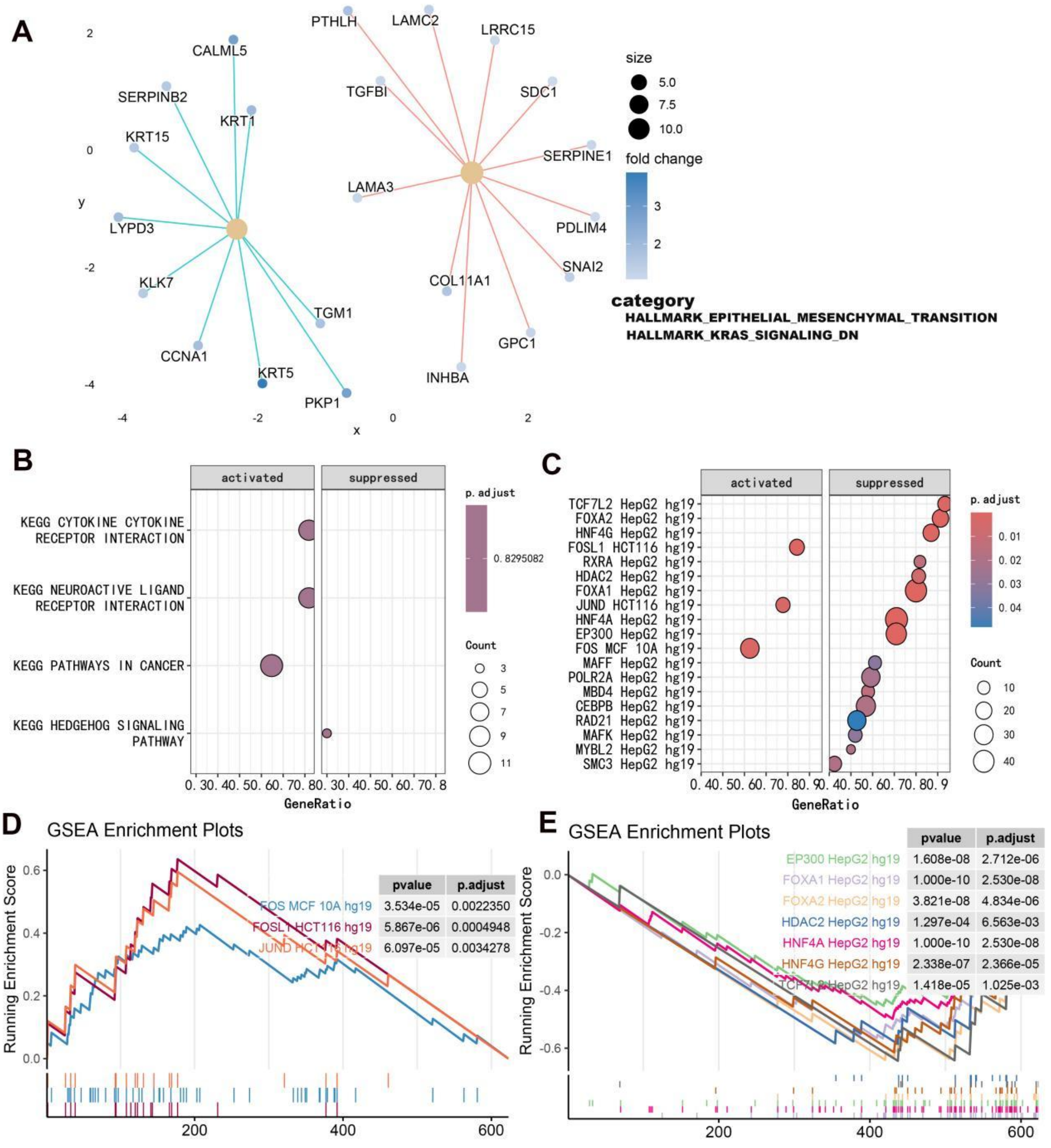

Supplement: Supplementary file 2 — Data S2: cam471605‐sup‐0002‐Supinfo2.pdf. [file CAM4-15-e71605-s001.pdf]
